# Multimodal imitative learning and synchrony in cetaceans: A model for speech and singing evolution

**DOI:** 10.3389/fpsyg.2023.1061381

**Published:** 2023-04-11

**Authors:** José Zamorano-Abramson, Maëva Michon, Ma Victoria Hernández-Lloreda, Francisco Aboitiz

**Affiliations:** ^1^Centro de Investigación en Complejidad Social, Facultad de Gobierno, Universidad del Desarrollo, Santiago, Chile; ^2^Grupo UCM de Psicobiología Social, Evolutiva y Comparada, Universidad Complutense de Madrid, Madrid, Spain; ^3^Centro de Estudios en Neurociencia Humana y Neuropsicología, Facultad de Psicología, Universidad Diego Portales, Santiago, Chile; ^4^Laboratory for Cognitive and Evolutionary Neuroscience, Department of Psychiatry, Faculty of Medicine, Interdisciplinary Center for Neuroscience, Pontificia Universidad Católica de, Santiago, Chile; ^5^Departamento de Psicobiología y Metodología de las Ciencias del Comportamiento, Facultad de Psicología, Campus de Somosaguas, Universidad Complutense de Madrid, Madrid, Spain

**Keywords:** multimodal imitation, vocal learning, synchrony, cetaceans, communication evolution, speech evolution, singing evolution

## Abstract

Multimodal imitation of actions, gestures and vocal production is a hallmark of the evolution of human communication, as both, vocal learning and visual-gestural imitation, were crucial factors that facilitated the evolution of speech and singing. Comparative evidence has revealed that humans are an odd case in this respect, as the case for multimodal imitation is barely documented in non-human animals. While there is evidence of vocal learning in birds and in mammals like bats, elephants and marine mammals, evidence in both domains, vocal and gestural, exists for two Psittacine birds (budgerigars and grey parrots) and cetaceans only. Moreover, it draws attention to the apparent absence of vocal imitation (with just a few cases reported for vocal fold control in an orangutan and a gorilla and a prolonged development of vocal plasticity in marmosets) and even for imitation of intransitive actions (not object related) in monkeys and apes in the wild. Even after training, the evidence for productive or “true imitation” (copy of a novel behavior, i.e., not pre-existent in the observer’s behavioral repertoire) in both domains is scarce. Here we review the evidence of multimodal imitation in cetaceans, one of the few living mammalian species that have been reported to display multimodal imitative learning besides humans, and their role in sociality, communication and group cultures. We propose that cetacean multimodal imitation was acquired in parallel with the evolution and development of behavioral synchrony and multimodal organization of sensorimotor information, supporting volitional motor control of their vocal system and audio-echoic-visual voices, body posture and movement integration.

## 1. Introduction

Language and music are one of the main human universals that define and set us apart from the rest of the animal kingdom, arising in every human society no matter what other cultural products are absent (Nettl, 2000; Hauser and McDermott, 2003; Patel, 2003; Fitch, 2006; Cross, 2009; Aboitiz, 2017). Speech and vocal music or “singing” are hallmarks of human evolution, both abilities relying on the capacity for motor imitation and vocal learning of complex pitch and rhythmic hierarchically organized sound structures (Patel, 2003). The central role of spoken language and singing in human sociality and the shared features between them suggest a common evolutionary origin (Brown, 2001; Aboitiz, 2017). In fact, the origin of both capacities is one of the biggest unsolved puzzles of human evolution, how our ancestors came to acquire the capacity for speech and singing are still poorly understood (Hauser and McDermott, 2003; Mithen, 2005; Fitch, 2006; Cross, 2009; Cross et al., 2013).

Darwin’s model of language evolution highlighted the crucial role of singing in the origin of human language, proposing that a critical step for speech evolution was the emergence of vocal imitation, which first was used “in singing true musical cadences” (this is currently called the “musical protolanguage hypothesis”; Fitch, 2013). Nowadays it is still not clear if music evolved from speech, or the other way around, or if the similarities between music and language arose independently by convergent evolution (Hauser and McDermott, 2003; Fitch, 2006; Cross, 2009; Cross et al., 2013). According to modern variations to Jespersen’s (1922) second model, namely, that both language and music were descendants of “half-musical” utterances, the shared features between music and language could have evolved first, and their domain-specific features evolved later as part of a branching process, making language and music homologous functions (Brown, 2000, 2001; Fitch, 2005; Cross et al., 2013).

Part of the difficulty in elucidating the possible evolutionary paths of human complex vocal communication origins and in determining what traits are shared between language and music, specifically speech with singing, is due to the fact that human language and music are both multicomponent phenomena based upon a diverse set of biological and cognitive mechanisms working together. Therefore, it is very likely that the diverse capacities emerged at different times in evolution and, accordingly, they probably served diverse functions that could have changed over time.

What is clear among these puzzles of capacities is the crucial role that social learning processes, particularly imitation, have played in the evolution of both capabilities. This capability is considered one of the most important adaptive benefits of sociality and the main prerequisite for the evolution and development of human social complexity, cooperation, culture and language (e.g., Heyes, 2009). Broadly defined, social learning entails acquiring knowledge about the animate and inanimate world (including the own social norms) that is influenced by observation of, or interaction with, another individual or its products (Galef and Laland, 2005; Heyes, 2012; Hoppitt and Laland, 2013). Learning to do things the way others do them rather than learning solely from one’s individual experience is an adaptive skill as it can reduce the costs and time and effort associated with individual trial and error learning (Boyd and Richerson, 1988; Heyes, 1994).

However, social learning is not a unitary process, and the diverse forms it takes can potentially be driven by different psychological processes (Caldwell and Whiten, 2002; Galef and Laland, 2005; Zentall, 2006; Heyes, 2012; Hoppitt and Laland, 2013). Taxonomies of social learning include, among other categories; stimulus and social enhancement, observational conditioning, response facilitation/contagion and imitation. This diversity of definitions and the cognitive mechanisms supposedly involved in each one, has made the comparative study of social learning a source of considerable debate in the behavioral sciences (Bates and Byrne, 2010). Among them, the notion of “Imitation” has given rise to disputes on issues such as what kind of mechanism deserves to be called genuine imitation, and if it is shared with some other animal species, that is, whether a non-human animal could have the ability to generate traditions that could be called “cultural” (e.g., Byrne and Russon, 1998; Tomasello, 2009; Bates and Byrne, 2010). In fact, in the past, the term “imitation” was used indiscriminately in a broad sense to refer to any of the social learning mechanisms above mentioned (Heyes, 2021). Nowadays, the term is mostly used in a narrower sense to refer mainly to one type of social learning in which an observer copies the ‘form’ or the topography of a model’s body movements; that is, how parts of the body move relative to one another (Heyes, 2021). If the behavior copied by the “observer” from the “demonstrator” is novel, that is, does not pre-exist in the observer’s behavioral repertoire, the terms “productive imitation” (Byrne and Russon, 1998), “complex imitation” (Heyes, 2012), or “true imitation” (Zentall, 2006), are often used. Importantly, this kind of imitation is thought to reflect the operation of complex cognitive processes (Byrne, 2002; Zentall, 2006).

In this article, we will discuss comparative experimental evidence from the bottlenose dolphins (*Tursiops truncatus*), the orca (*Orcinus orca*) and the belugas (*Delphinapterus leucas*), among the few mammalian species that have been reported to display multimodal imitative learning besides humans, where “productive” vocal and motor imitative cognitive skills are present.

First, we will contextualize and distinguish multimodal imitation within the different taxonomical categories of social learning that exist. We also discuss these differences in terms of the supposed simplicity or complexity of the cognitive mechanisms that underpin each one. Then we will review the experimental evidence of multimodal imitation in cetaceans, and their role in sociality, communication and group cultures. We will also depict some similarities and particularities of their perceptual and communicative systems, with special emphasis on echolocation and synchrony. After, we will discuss what has been proposed for the development and evolution of human imitation.

Overall, this review will contribute to substantiating the importance of multimodal imitative learning as a central mechanism in the acquisition and temporary stability of cetacean group cultures. Finally, based on this comparative evidence, we propose a model for the development of the multimodal imitation capacity in cetaceans alongside the evolution of their behavioral synchrony and multimodal organization and its possible convergence with human linguistic and musical communication evolution.

## 2. Dissecting the imitation capacity

### 2.1. Correspondence problem and complexity

Bodily motor imitation is generally believed to be a cognitively demanding form of social learning, as it “requires the cognitive system to know that the model’s and observer’s actions are similar from a third-party perspective; that it must be able to transform a sensory representation of the model’s body movements into a motor representation controlling the observer’s action” (pp R228 Heyes, 2021). This constitutes one of the main problems raised by imitation, that is, how these sensorimotor associations are established and by means of which neurocognitive mechanisms. This problem is known as the “correspondence problem” and it is still unresolved in the scientific community.

### 2.2. Transparent vs. opaque, transitive vs. intransitive

In this vivid “correspondence problem” debate, one of the distinctions that has attracted most attention is the difference between copying of “transparent” actions, when an observer performs the same movement, they will see the same spatial configuration. However, when performing “opaque” actions, the observer may see a different spatial configuration, or may not be able to see their own action at all, such as with facial expressions or whole-body movements like bowing or joining hands behind the back (as described by Heyes, 2021). It has been hypothesized that the former engages cognitive skills that can be less demanding than those required to match opaque actions, as in the latter what makes imitation a more difficult achievement is the difference between the information that is available to the observer’s senses when the body movement is performed by the model and when it is performed by the observer (Zentall, 2006; Heyes, 2021). Similarly, some researchers have argued that the copying of so-called transitive (object-oriented) actions is likely to engage cognitive skills that can be different from those required to match intransitive (body-oriented) actions (Heyes and Ray, 2000; Heyes, 2001). It has been suggested that intransitive imitation is limited to humans and the great apes (Miles et al., 1996). However, after several studies using this procedure in chimpanzees (Hayes and Hayes, 1952; Tomasello et al., 1993; Custance et al., 1995; Myowa-Yamakoshi and Matsuzawa, 1999) and orangutans (Call, 2001), it remains controversial whether great apes in general demonstrate a capacity for matching of their own body part actions to those of another ape (Whiten et al., 2009). In sum, comparative evidence has shown how difficult-but not impossible-it is for animals to copy pure movements that have no environmental effects compared with object-related actions.

### 2.3. Imitation of novel actions (“true imitation”; “imitative learning”; “production learning”)

Alternative and even narrower definitions have been proposed for imitation, for example arguing that the only form of true imitation that is found on higher-level cognitive skills, called “production learning,” is when the action of the model is entirely “novel” to the observer (Zentall, 2003, 2006; Hoppitt and Laland, 2013; Heyes, 2021). However, it is important to note that when defining novelty, there will always be some degree of similarity to what the observer has done before (Whiten et al., 1996) and that the imitation of novel actions could still be possible by the same learning mechanisms as familiar actions (see Heyes, 2021 and Michon et al., 2022 for review).

In any case, as can be seen in all the definitions mentioned above, the vast majority of them are oriented toward defining imitation in the “motor” or “gestural” domain (copying the topography of body movements), where as a result of observation of the model, the observer moves its body parts similarly to the model’s body parts (see Heyes, 2021). Moreover, it has been argued that the faithful reproduction of a demonstrator’s behavioral topography *via* imitation plays a key role in the emergence of human technology (Tennie et al., 2017) as it enables the accumulation of modifications over time (i.e., the ratchet effect) facilitating the evolution of culture together with other forms of social learning (Tomasello, 1999). Alternatively, it has been proposed that motor imitation’s crucial role relies on the cultural inheritance of human gestures, dialects, languages, and rituals (Heyes, 2021).

### 2.4. Vocal learning

Vocal imitation (also referred to as vocal production learning) arises when an animal learns to modify its vocal output to match signals of other individuals. This includes the production of novel signals or modifications of signals that were already in an animal’s repertoire (Janik and Slater, 1997, 2000). Therefore, similar to bodily motor “productive” imitation, “true” or “complex” vocal learning is defined as the ability to copy sounds that are not part of the normal species-specific repertoire (Janik and Slater, 1997, 2000), which implies that the subject must learn a new acoustic template for the vocalization and then learn to develop a vocalization that matches the template (Tyack, 2020). Recent evidence have extended this definition as follows: vocal production learning is the production of modified or novel vocalizations, as a result of learning from the perceptual experience of the acoustic signals of others (Vernes et al., 2021).

Importantly, most researchers consider vocal learning as less complex since the copy of a sound may not involve the correspondence problem, even when the sound is novel (Heyes, 2021). This argument usually relies on the concept of “equimodality,” that is, that when you hear someone producing a sound, you perceive it using the same hearing device used to perceive the sounds you produce yourself. On the other hand, the copy of motor actions, particularly facial gestures, requires “visual-tactile (and proprioceptive) cross-modal performance” (see Shettleworth, 2010). In other words, seeing an action being performed recruits a different set of sensory systems to those recruited to sense the actions being performed by your own body. However, we do not necessarily agree with this view as in the case of hand movements (and to some degree body movements), the observer may use the same visuo-motor circuits to monitor the observer’s and the own movements, that may be comparable to the audio-vocal circuits involved in vocal imitation; and in both cases proprioceptive signals can be relevant for successful copying (see Hickok, 2022). Moreover, this view of vocal imitation as “less complex” is somewhat surprising since this capacity is a hallmark of human spoken language, which, along with motor imitation and other advanced cognitive skills, has certainly fuelled the evolution of human culture. In addition, cross-modal interaction between gestures (especially facial gestures) and vocal communication is highly relevant in speech development and most likely it was so in the evolutionary origins of speech (Michon et al., 2022).

Finally, the capacity for vocal learning is not strictly a gift given to some animals, but as said there are different levels of vocal learning abilities in different species. Petkov and Jarvis (2012) categorized species on the basis of their vocal learning capacities, where animals like monkeys display a limited learning capacity, songbirds and parrots are relatively complex vocal learners and our species apparently exceeds all the others in the voluntary and fine control of phonation (Petkov and Jarvis, 2012).

In fact, and strikingly, comparative evidence has revealed that humans are an odd case among mammals, as the case for vocal production learning exists mainly in birds (see Catchpole and Slater, 2003), with only a few instances in mammals, with confirmed evidence for elephants (Stoeger et al., 2012), bats (Knörnschild et al., 2010), pinnipeds (Ralls et al., 1985; Schusterman, 2008; Reichmuth and Casey, 2014) and cetaceans (Tyack and Sayigh, 1997; Janik, 2014). Likewise, current evidence supports that vocal imitation is harder than motor imitation for primates, as Japanese monkeys seem to require greater effort in motor preparation for voluntary control of vocalization than for manual actions (Koda et al., 2018). Even after training, the experimental evidence for imitation is scarce, with reports for spontaneous imitation of human whistles and of human “wookies” in orangutans under human care (Wich et al., 2009; Lameira et al., 2016) and novel atonal-breathing utterances in Koko, the famous female enculturated gorilla (Perlman and Clark, 2015).

On the other hand, while there has been little evidence of vocal imitation in monkeys and apes in the wild (Hauser et al., 2002), more recent reports indicate that wild apes may have a greater degree of vocal plasticity than has been usually considered, with social-dependent vocalization patterns in orangutans and combinatorial vocal sequences in chimpanzees (Girard-Buttoz et al., 2022). Furthermore, the new-world marmoset monkeys have a complex social and vocal behavior, displaying reciprocal turn-taking vocalizations and capacity for vocal learning from adults during development (Margoliash and Tchernichovski, 2015; Pomberger et al., 2019; Gultekin et al., 2021; Varella and Ghazanfar, 2021); however, it is not clear yet whether this may qualify as imitation in the sense used in this article. Still, there is the apparent paradox of how can vocal imitation be difficult for many primates, if the correspondence problem is supposed not to exist in this modality? This fact has not only risen the open question of whether it is adequate to claim that vocal imitation is less complex than motor imitation, but also what kind of mechanism (motor, auditory or and/or cognitive) has driven its evolution (see Feenders et al., 2008; Aboitiz, 2017). For example, it has been suggested that the evolution of vocal learning is driven by modifications of the motor rather than the auditory system, which is more conserved across species, and partly relies on general-purpose mechanisms (see Aboitiz, 2017 Chapter 9; Feenders et al., 2008; Petkov and Jarvis, 2012).

Comparative evidence exploring both modalities, vocal and gestural learning, is needed to clarify if the same happens in other vertebrates and specifically in other mammal species. Moreover, these results evidence that if vocal imitation is infrequent in non-human mammals, the capacity for them to imitate in both domains, bodily and acoustic, may be even scarcer. The evidence regarding diversity in manual and vocal control abilities in different primates supports that in the human lineage, vocal evolution probably evolved in close coevolution with hand control, associated to tool making and gesture behavior (Aboitiz, 2017, 2018; Koda et al., 2018). Importantly, recent neurological evidence in baboons has shown a dissociation of handedness’ types between manipulative action and communicative gestures, with only the latter being associated with a frontal cortex lateralization signature which might reflect a phylogenetical continuity with language-related Broca lateralization in humans (Becker et al., 2022). Perhaps the conjunction of vocal and motor communication systems in early Hominins drove the development of multimodal imitation capacities leading to the origin of a rudimentary proto-language, using both vocal and gestural signals. In this line, perhaps marmoset monkeys’ vocal abilities might better resemble the early vocal behavior of ancestral hominins than the macaques’. Basic vocal learning capacities used in turn-taking vocal interactions, perhaps comparable to those of marmoset monkeys (Hage et al., 2016; Takahashi et al., 2016), could have evolved early in our human ancestors, which complies with the apparent antiqueness of the human-specific mutations of the speech-related gene FOXP2 in our lineage (Aboitiz, 2018; Atkinson et al., 2018). A degree of voluntary control of vocalization for social purposes, together with skilled hand control associated with early tool-making abilities, and more specifically, for gestural expression, may have provided the feedstock for the evolution of visual-gestural and auditory-vocal imitation capacities that distinguish our species.

## 3. Cetacean imitation

Similarly to great apes and other highly social terrestrial mammals, cetaceans exploit socioecological niches that have been selected for some convergent adaptations such as long lifespan, large brains and high encephalization quotients (Yurk et al., 2002; Marino et al., 2007). They have been depicted as highly sociable, with a complex, organized and cooperative sociality and endowed with advanced cognitive skills (see Marino, 2022) with unique group-specific behavioral signatures including vocal repertoires and hunting and foraging tactics that do not seem to be either ecologically determined or genetically inherited (Deecke et al., 2000; Yurk, 2003; Riesch et al., 2012; Krützen et al., 2014). In fact, cetaceans are often presented as representative species of potential non-human cultural traditions with group-specific vocal repertoires and motor behaviors (Rendell and Whitehead, 2001; Whitehead and Rendell, 2014). Accordingly, it is not surprising that they are another of the mammalian groups that are often mentioned in reviews of imitation research for possessing cognitive abilities comparable to those of great apes (Herman, 2002, 2006, 2010; Yeater and Kuczaj II, 2010; Marino, 2022).

### 3.1. Evidence from ecological observations in the wild

Although the existence of social traditions does not prove imitative learning *per se*, in cetaceans there have been observational reports of specific behavioral tactics in several species in the wild, which exhibit substantial behavioral diversity between sympatric groups in terms of the motor and acoustic features of their behavioral repertoires (foraging tactics, songs, calls, or dialects among others; see Rendell and Whitehead, 2001 for review).

With regards to baleen cetaceans the most documented case belongs to the humpback whale, with motor behaviors candidates to be learned by imitation. For example, some foraging methods consist of complex cooperative herding that includes bubble-cloud feeding combined with trumpet-like calls and “lobtail feeding” (see Whitehead and Rendell, 2014 for review). However, the most well-known imitation candidates are the whale’s songs, which possess several features that make them one of the most complex vocal displays in the animal kingdom: (a) they display hierarchical structures where sequences of units (discrete sounds) conform phrases that are repeated several times, each set of repetitions of a phrase type conforming a theme and a sequence of different themes conforming a song (units → phrases → themes → songs; Payne and McVay, 1971); (b) they are long, lasting from 6 to 35 min and can be repeated continuously during a song session (e.g., 22 h); (c) they undergo constant evolutionary change. This last characteristic is most remarkable as it constitutes a coordinated change over time, where all males in a population tend to share the same song, even while it changes gradually throughout the reproductive season (which results in that the shared song at the start and at the end of the season is not the same). This shared song that slowly drifts in its structure over time is almost unique among non-human mammals and the most accepted hypothesis is that other whales might learn novel variations introduced by other individual, however alternative explanations exists (see Iii, 2018).

Toothed cetaceans, also known as odontocetes, include species such as bottlenose dolphins and orcas that are thought to have their own unique and potentially culturally transmitted traditions. This could be the result of social learning through mechanisms like motor and vocal imitation. Similarly, these reports include foraging tactics as bottlenose sponging, shelling fishing tactics, mud-bank ring feeding (or mud plume fishing) and herding cooperative feeding (see Krützen et al., 2014). Additional examples are the orcas’ intentional beaching (Lopez and Lopez, 1985; Guinet, 1991; Guinet and Bouvier, 1995), the “carousel feeding” technique (Similä and Ugarte, 1993), or the “cooperative wave-washing behaviour” to take seals off the ice floe (Visser et al., 2008; Pitman and Durban, 2012) among others.

Although most of the authors are inclined to believe that these behaviors, reported in the wild, could be largely scaffolded by social learning processes including motor and vocal imitation (Ford, 1991; Deecke et al., 2000; Yurk et al., 2002; Barrett-Lennard and Heise, 2006; Weiß et al., 2011), other scholars have argued that the evidence for imitation is weak at best and that their ‘cultural traditions’ can be explained by other less complex learning processes (Caro and Hauser, 1992; Galef, 2001). Therefore, to elucidate the social learning mechanisms that underpin these group-specific behaviors, we will focus our review on experimental studies realized in controlled conditions that rule out alternative explanations.

### 3.2. Evidence from controlled experimental settings

While there are several procedures to study imitation in animals, the most used (or the gold standard) in cetaceans in controlled conditions is the “Do as I Do” method. This paradigm, initially used by Hayes and Hayes (1952) in a study of motor imitation in a home-raised chimpanzee, involves copying another’s action under a specific signal (“Do this!”), without any other scaffolding information (e.g., results-based cues). This specific method consists of training the observer to respond to a visible gesture-based command “copy” (“Do that”) given by the trainer. The sign itself could be gestural (usually hands) or vocal. This generic “copy” sign command made up for this purpose is the same over all presentations or trials, regardless of the behavior from the model that must be copied by the observer. Some authors have argued that to solve this task the observers need to “understand” that he/she is required to imitate what the model is performing, that is, that the animal subjects need to have some kind of concept of imitation, as the method depends on the generalization of a trained signal to a conceptual order that is “copy what I am (or what the other is) doing” (Whiten, 2000; Herman, 2002, 2006, 2010). This training technique has been successfully used in several species of great apes (Tomasello et al., 1993; Custance et al., 1995; Call, 2001), dogs (Topál et al., 2006; Fugazza et al., 2016), cats (Fugazza et al., 2021) and cetaceans: dolphins (Xitco, 1988; Bauer and Johnson, 1994; Herman, 2002), orcas (Abramson et al., 2013, 2018) and belugas (Abramson et al., 2017).

#### 3.2.1. Motor imitation

Experimental evidence for the ability of motor imitation in cetaceans has been demonstrated mainly in the bottlenose dolphin (Xitco, 1988; Bauer and Johnson, 1994; Herman, 2002; Kuczaj and Yeater, 2006). Xitco (1988) reported that dolphins can be trained using the “Do as I do” method to imitate another dolphin or even a human on command for familiar as well as for novel behaviors. They were even capable of imitating familiar behaviors of a conspecific after a delay of up to 80 s. Although some authors suggest that any kind of imitation could be viewed as a “delayed synchrony,” the term “delayed (or deferred) imitation” refers to the ability to encode, store and retrieve the memory of a perceived action and then use it as the basis to reproduce the model’s action after a sufficiently long delay (1 min or more) that excludes the kind of reflexive response based on short-term memory commonly thought to be responsible for immediate imitation (Cowan et al., 1997). It is important to note that this deferred imitation condition in the dolphins involved some intransitive actions which contributed to rule out response facilitation, as no environmental cues were present (other than the previous action of the demonstrator itself) when the copy command was given. Bauer and Johnson (1994) extended these results by training two naïve dolphins to imitate a set of familiar and two completely novel behaviors on command, although neither dolphin imitated the novel behaviors. In other studies, dolphins also showed the ability to “repeat” the last behavior they produced themselves, which could potentially be seen as imitation of their own actions (Mercado et al., 1998). Dolphins also were able to perform a variety of familiar and uninstructed new behaviors created by themselves together in close synchrony both in timing and in characteristics (Herman, 2002; Kuczaj and Yeater, 2006), and were also able to imitate the behaviors of another dolphin in a blindfolded (i.e., wearing eyecups) condition (Jaakkola et al., 2010). Interestingly, the blindfolded echolocation dramatically increased when copying a human as compared to other dolphins, suggesting that the subject actively switched between strategies: recognizing behaviors *via* characteristic sounds when possible, but *via* echolocation for the more novel sounding behaviors of the human. Such flexibility in changing perceptual routes demonstrates that the dolphin’s imitation was not automatically elicited, but rather results from an intentional, problem-solving approach to imitation (Jaakkola et al., 2013). In the last decade, experiments performed on two other cetacean species, orcas and belugas have strengthened these results.

Three orcas trained with the same “Do-as-Other-Does” paradigm used in dolphins were able to copy 100% of the demonstrator’s novel actions, with 2 novel behaviors (out of 2) that achieved full matches in the first trial in one subject. This study provided experimental evidence for body production imitation in orcas that is comparable to that observed in dolphins tested under similar conditions (Abramson et al., 2013). Remarkably the subjects learned the copy command signal “Do that” very quickly, that is, 20 trials on average. Taken together, these results suggest that orcas may be particularly skilled in matching others’ actions (see Yurk, 2003; Abramson et al., 2013). This finding has been supported by a recent study on deferred imitation of intransitive actions in two subjects of this species (Zamorano-Abramson et al., 2023). The subjects observed a demonstrator’s target actions and then were requested to reproduce them after a delay interval ranging from 60 to 150 s. Importantly, the experimental design also included the interspersal of distractor (non-target) actions performed by the demonstrator and by the subjects during the retention interval. The subjects copied the model’s target actions in all conditions. These findings rule out the operation of simple automatic social learning mechanisms as explanations for their behavioral matching and suggest instead that the subjects were able to keep in memory and then retrieve an enduring representation of the demonstrated action, flexibly and selectively, and to use it to match their response. They also lend further support to the proposal that the subjects’ performance relied not only on a mental representation of the specific actions that were requested to copy, but also flexibly on the abstract and domain general rule requested by the specific “copy command.” Orcas are known to be highly social, they display behavioral coordination, and learn socially from their group’s members through contextual and production imitation (Yurk, 2003; Abramson et al., 2013). This study strengthen the view that orcas and other cetaceans are capable of flexible and controlled social learning (Zamorano-Abramson et al., 2023).

Finally, using this “Do as other does” paradigm in belugas, the first evidence of contextual imitation of intransitive (body-oriented) motor movements for this species was provided. Here, it has reported that beluga whales are capable of copying conspecific’s familiar intransitive and opaque body movements on command (Abramson et al., 2017). This finding indicates that beluga whales, similar to dolphins, orcas, dogs and apes, can be trained to imitate actions on command in a “do-as-I-do” task. Recently, a follow up of this experiment proved that another beluga was also capable of imitating novel intransitive and opaque body movements on command (Zamorano-Abramson et al., 2023). The current balance of evidence therefore supports the notion that three species of toothed cetaceans are capable of motor imitation including novel behaviors or true imitation in bottlenose dolphins, orcas and belugas (see Table 1).

**Table 1:** Summary of the main experimental results on cetacean imitation of novel motor and vocal behaviors and other related complex imitative capacities.

Modality	Timing	Model	Experimental Procedure	Species		
Vocal				Dolphin	Orca	Beluga
	Consecutive					
		Human	Spontaneous			Ridgway et al. (2012)
			Controlled	Lilly (1965)	Abramson et al. (2017)	Murayama et al. (2014)
		Conspecifics	Spontaneous	Reiss and McCowan (1993)	Crance et al. (2014)	
		Heterospecifics	Spontaneous	Favaro et al. (2016) (*Risso’s dolphin* *copying Bottlenose dolphi*ns)	Musser et al. (2014) (*Imitation of Bottlenose dolphins*)	
					Foote et al. (2016) (*Imitation of sea lions*)	
		Synthetized	Controlled	Richards et al. (1984) and Richards (1986)		
Motor	Synchronic	Conspecifics	Controlled	Herman (2002)		
	Consecutive	Human		Xitco (1988) and Herman (2002)		
				Jaakkola et al. (2013) *Blindfolded*		
		Conspecifics	Controlled	Xitco (1988) and Herman (2002)	Abramson et al. (2013)	Abramson et al. (2017)
				Jaakkola et al. (2010) *Blindfolded*		
				Jaakkola et al. (2013) *Blindfolded*		
	Differed		Controlled	Xitco (1988) and Herman (2002)	Zamorano-Abramson et al. (2023)	

#### 3.2.2. Vocal imitation

Although knowledge about marine mammal imitative vocal abilities is still poor, the available information reported above indicates that there may be significant cross-species variability with several species showing remarkable vocal flexibility. For example, in the case of pinnipeds, there is the well-known case of *Hoover*, a captive adult male harbor seal who was able to spontaneously utter about 12 human words (including the tone, the intensity or the accent of a specific individual; Ralls et al., 1985). In the same line, recent reports have shown flexible vocal behavior evidence in the harbor seal pups as they can modified their vocalizations by shifting their fundamental frequency to cope with increased noise (Torres Borda et al., 2021). In contrast, sea lions have failed to exhibit vocal imitation and their vocal flexibility has been found to be poor (see Schusterman, 2008; Reichmuth and Casey, 2014 for review), despite decades of research.

In cetaceans, evidence under controlled experimental conditions for vocal imitation ability has been documented mainly in the bottlenose dolphin. Lilly (1965) reported the first observations of spontaneous vocal imitation in a cetacean, including some elements of human words. Current experimental evidence shows that bottlenose dolphins can imitate novel sound patterns (Reiss and McCowan, 1993), and can spontaneously imitate computer-generated artificial sounds, and use them even to label objects (Richards et al., 1984; Richards, 1986). In addition, it has been demonstrated that this species can mimic the calls produced by another group member effectively addressing the whistle owner, technically referred to as signature whistles (Caldwell and Caldwell, 1968). Therefore, this suggests that vocal production learning is the main mechanism involved in the development of these recognition signals (Janik and Slater, 1997, 2000; Janik, 2014).

Another cetacean species that has shown the ability for vocal production learning is the beluga. Recently, Ridgway et al. (2012) reported that a beluga spontaneously imitated human speech-like sounds. These findings have been supported by an experimental study in which one subject was actually capable of imitating some components of human vocal sounds. Here, Murayama et al. (2014) tested the ability of a male beluga to copy familiar conspecific sounds, novel artificial (computer-generated) sounds and human speech. They found that their study subject succeeded at imitating both familiar and novel sounds. This spontaneous mimicry of vocal signals during interactive situations and cognitive tests suggests that these two species (bottlenose dolphins and belugas) possess both cognitive flexibility and the drive to engage in cross-species communication (Herzing, 2015).

An adventitious cross-fostering study of a Risso’s dolphin raised in a group of bottlenose dolphins showed that the cross-socialized animal produced vocal signals more akin to those of its bottlenose pool-mates than those of Risso’s dolphins in the wild (Favaro et al., 2016). In similar accidental or natural cross-socializing experiments documented in the wild and in controlled settings it has been reported that orcas can copy novel calls from conspecifics (Bain, 1986; Crance et al., 2014), and even from heterospecifics such as bottlenose dolphins (Musser et al., 2014) or sea lions (Foote et al., 2006). Finally, these opportunistic reports have been supported by experimental evidence in this species showing the ability to also imitate novel vocalizations from another orca (vocal imitative learning) and even from human speech (vocal mimicry; Abramson et al., 2017). Here, the subject made recognizable copies of all familiar and novel conspecific and human sounds tested and did so relatively quickly (most during the first 10 trials and three in the first attempt). These results lend support to the hypothesis that the vocal variants observed in natural populations of this species can be socially learned by imitation.

In sum, three species of cetaceans, namely bottlenose dolphins’, orcas and belugas have demonstrated the ability for imitation of novel complex behavioral patterns in both motor and vocal domains (see Table 1). As far as we know, no other non-human animal species tested to date has shown this capacity at this complex state. It is likely that some other cetacean species and vocal learning mammals not yet tested in both conditions (like bats and elephants) may share similar multimodal imitative abilities due to their similar evolutive socio-ecological pressures.

#### 3.2.3. Multimodal imitation

Taking into account all the distinctions mentioned so far, by way of consensus we are going to define multimodal imitation in a narrow sense as it follows: the ability for copying novel bodily (kinesthetic motor patterns without producing any kind of vocalization) and vocal productive patterns in both domains. However, we have discussed the vivid debate about what constitutes a novel behaviour, and the unclear features for identification and measure of the “simplicity” or “complexity” of the cognitive mechanisms that underpin this and other kind of social learning process. Therefore, we will complementarily discuss other types of social learning that are also commonly referred to as “complex,” such as the copy of intransitive actions, and self, synchronic and deferred imitation.

Surprisingly, besides cetaceans, non-human multimodal imitation has been confirmed only in Psittacine birds (specifically in budgerigars and a grey parrot; Galef et al., 1986; Moore, 1992; Farabaugh et al., 1994; Hile et al., 2000). Interestingly, it has been recently reported that multimodal (visual and auditory) exposure to a tutor is relevant for birdsong vocal learning (Varkevisser et al., 2022). Paradoxically, evidence for vocal learning, which supposedly is less complex than motor imitation, has been widely studied in birds but much less in non-human mammals. Therefore, the few mammals that can be candidates for multimodal imitation are the instances that we already know, namely elephants (Stoeger et al., 2012), bats (Knörnschild et al., 2010), pinnipeds (Ralls et al., 1985; Schusterman, 2008; Reichmuth and Casey, 2014) and cetaceans (Janik, 2014). As far as we know, besides toothed cetaceans, no experimental studies of motor productive imitation have been done in most of these species, so the question remains open for them and leaves cetaceans as the only non-human mammals in which both domains have been tested with positive results. Gopnik and Meltzoff (1993) suggested that kinesthetic imitation rests on a cross-modal representational system that encodes information about one’s own behaviors in the same way that it encodes information about others’ behaviors. As a result, the system allows representations of others’ behaviours and representations of one’s own kinesthetic sensations to be mapped onto one another. In the case of cetaceans and other human-acquainted animals that have just observed the command to imitate an action, the repetition or imitation of an action requires to match their own (internal) system with an external motor-gestural pattern or sequence (because it comes from the model) which they access by vision (or hearing in the case of vocalizations). This capacity is based on the multimodal reactivation of this episodic somatosensory information (probably stored in short-term memory). For example, in the case where dolphins were required on command to repeat their own object-action sequences to non-specified objects (Mercado et al., 1998, 1999), a multimodal representation of ongoing events and a flexible capacity to remember specific details of those events has been proposed to account for the findings (see Baddeley, 2000; Mercado and DeLong, 2010). That is, multimodal representations are used to support the selection and production of actions as well as mental simulations of actions and events (Barsalou et al., 2003; Garbarini and Adenzato, 2004; Barsalou, 2008). This type of multimodal representations has been identified as the core of higher-order same/different concepts in humans. For example, Cook and Qadri (2021, p. 98) define them as concepts or rules that are “transferable or applicable across all manner of perceptual (i.e., low-level) and conceptual stimulation, resulting in a higher-order abstraction that is independent of modality.”

In cetaceans, this has been supported by Murayama et al. (2017) who reported that a beluga whale was capable of spontaneous establishment of cross-modal stimulus equivalence between visual and auditory symbols (novel objects that were untrained). It is important to note that until this study the capacity for cross-modal stimulus equivalence was considered exclusively human and attributed to the linguistic faculty (Michon et al., 2022). Similarly, the results on deferred imitation in orcas mentioned above, which also include distractions during the retention interval before the copy command was given, gives further support to this interpretation (Zamorano-Abramson et al., 2023). Therefore, Gopnik’s notion of a cross-modal representational system fits well not only with the discovery of mirror neurons in rhesus monkeys (Rizzolatti et al., 1996) but also with the echolocation system of toothed cetaceans and its possible evolutive relation with their capacity for multimodal imitation, in both the vocal and the motor domain.

## 4. Discussion

### 4.1. Cetacean multimodal imitation and communication

Cetaceans have developed several anatomical and biochemical hearing and vocal specializations that allow them to hear and emit a much wider range of sound frequencies (into the ultrasonic range) than humans. Accordingly, the ability of cetaceans to explore and interpret their world *via* a sophisticated echolocation or biosonar should be carefully considered. Toothed cetaceans and bats emit short-broadband ultrasonic clicks vocalizations (“nasalizations” would apply better in this case) to localize prey and to explore their surroundings (Au, 1993). The information is obtained *via* analysis of echoes generated from objects by sound reception through a region of the lower jaw and transmission into the ear. It has been documented that dolphins change the temporal and spectral features of their echolocation signals in relation to distance, size, shape and acoustic properties of the focused target (Au, 2004; Jensen et al., 2009). That is, odontocetes and possibly mysticetes (see humpback whale songs long-range sonar hypothesis by Iii, 2018) can associate information that they receive from vision with information that they obtain from echolocation when this information concerns stationary objects, but they are even able to integrate dynamic information about movement across echolocation and visual senses. This means that echolocation *per-se* is a cross modal perceptual system as it is based on “active production and perception of an acoustic beam transformed to a visual cross-modal representation.”

Moreover, it has been suggested that echolocation in cetaceans not only plays a physical function of sensing the environment for one individual but also plays a social function in communication with other individuals. Experimental evidence shows that it is possible for an eavesdropping dolphin to discern object information from the returning echoes generated by the echolocation signals of conspecifics (Gregg et al., 2007; Arranz et al., 2016). All these results in cetaceans parallel the evidence of a convergent evolution of echolocation and vocal learning in bats. In addition, in bats, echolocation also facilitates communication in various forms, such as eavesdropping, territorial defense and courtship (Knörnschild et al., 2012).

But echolocation is not the only sophisticated adaptation we have to consider for the presence of multimodal imitation in aquatic animals. Terrestrial animals rely upon voice cues to encode individual identity with their vocalizations. Whereas the recipient must learn to recognize the voice of each individual, the speaker is not necessarily constrained to learn to produce voice cues. Speaker-specific cues stem from natural differences in the air-filled vocal tracts of each person. Diving mammals are unable to rely upon such vocal cues for individual recognition because, as they dive, the volume of gases in their vocal tract is halved with each additional atmosphere of pressure: this change in the vocal tract renders voice cues unreliable (Tyack, 2000). Therefore, diving mammals that rely upon individual-specific social relationships must learn to control their vocal apparatus to produce individually distinctive vocal signature signals with an individually distinctive frequency pattern like the signature whistle above mentioned. This may be another significant reason behind cetacean capacity for vocal learning.

While on one hand, the dark and opaque underwater environment may be a good reason to think of cetaceans as predominantly vocal creatures (with their echolocation sense being one of their more sophisticated adaptations), on the other hand we are rarely aware of the importance of their sense of touch and kinesthetic in their communicative systems in such environment (see Kremers et al., 2016). It has been suggested that cetaceans utilize a scale-dependent, multimodal sensory system to assess and increase prey encounters (Torres, 2017). Moreover, from its early years to the present day, research in the field of cetacean communication have evidenced how both, motor behavior and sound, correlate in several contexts (see Herzing, 2015 for review).

Regarding their sense of sight, while it is true that the marine environment limits visual perception, especially as depth increases, in clear waters light can penetrate up to 200 m and depending on turbidity, the range of visibility may vary from centimeters to tens of meters (see Lammers and Oswald, 2015). In fact, cetacean sense of sight is pretty good and more developed (or conserved from their terrestrial ancestors) than commonly thought (Kremers et al., 2016). Accordingly, they use it in a variety of contexts, mediating prey capture and social interactions (see Kremers et al., 2016 for review). Regarding short-range communication dynamics, cetaceans use a variety of visual displays where postures are thought to signal intent and demeanor of the signal emitter (Würsig et al., 1990; Dudzinski, 1996) for example in reproductive contexts when dolphins present their genital region to sexually attract a mating partner (Tyack, 2000) or as indication of the affiliative bonding between individuals reflected by proximity and synchronous movements (Connor, 2007). They also use vision for the inspection of objects above water (Madsen and Herman, 1980), sometimes surfacing vertically and lifting the head out of the water to do it (*spyhopping;* e.g., Ford, 1984; Whitehead and Weilgart, 1991). Similarly, dolphins have shown understanding of human pointing gestures produced above the water (Herman et al., 1999; Xitco et al., 2001). It is important to note that most of the motor imitation studies reviewed, use conspecific or human actions in which most of the topography (or even all of it in the case of human models), has been demonstrated above the surface, which clearly requires good vision (Abramson et al., 2013, 2017, 2018; Herman, 2002. Bender et al., 2009).

### 4.2. Cetacean multimodal imitation and synchrony

#### 4.2.1. Behavioral synchrony

Synchronous behaviors occur when two or more animals display the same behavior at the same time (Herzing, 2015). Synchronized behaviors in cetaceans like surfacing, breathing, swimming and diving has been documented in several communicative contexts. They have been hypothesized to play a role in affiliative processes as well as anti-predatory responses, allowing for close proximity and rapid coordinated response of individuals, with the multiple functions of showing affiliation and reacting to disturbance (see Senigaglia et al., 2012). Accordingly, their assessment has been considered an indicator of social relationship (Fripp et al., 2005) and alliance membership (Connor et al., 2006). For example, among dolphins these behaviors include collaborative activities as spinner dolphin behavior while dispersing from bays (Brownlee and Norris, 1994), surfacing behavior of Indian Ocean bottlenose dolphins (Connor et al., 2006; Sakai et al., 2010), and during herding behavior of females (Connor et al., 1992). Dolphins also display synchrony during defensive activities, as observed in male pantropical spotted dolphins while being herded into tuna nets (Pryor and Kang-Shallenberger, 1991) and in aggressive contexts, including interspecific interactions (Cusick and Herzing, 2014), and intraspecific aggression (Herzing, 2015).

Synchronized behaviors have been documented among other cetaceans: synchronized breathing cycles during resting and feeding behavior in killer whales (Ray,1986; Heimlich-Boran, 1988); synchronization of breathing in long-finned pilot whales (Senigaglia and Whitehead, 2012), diving cycles of sperm whales (Whitehead, 1996); foraging and diving behavior of mothers and calves in humpback whales (Tyson et al., 2012; Huetz et al., 2022) and adult lunge feeding characterized by controlled opening of their mouth in synchrony with strong flipper strikes (Simon et al., 2012). Recently, it has been reported that deep diving Blainville’s and Cuvier’s beaked whales show an extreme group synchronicity in their diving and vocal behavior, which can reduce their detection by killer whales (Aguilar de Soto et al., 2020). It is important to note that while this synchronization plays a role in reducing predation risk, it may also become maladaptive, as occurs in this and other cetaceans in cases of mass strandings induced by man-made predator-like sonar sounds (Aguilar de Soto et al., 2020). A striking case of behavioral synchrony in cetaceans is the “cooperative and coordinated wave-washing behaviour” displayed by Antartic type B, orcas to take seals off the ice floe (Smith et al., 1981; Visser et al., 2008; Pitman and Durban, 2012). This behavior is characterized by 2–7 animals abreast coordinated side-by-side swimming in a “tightly synchronized” formation to the point of deliberately generating a wave capable of breaking the floe and dragging the seal into the water (Visser et al., 2008; Pitman and Durban, 2012). According to Pitman and Durban (2012) “as they charged underwater toward the floe, the whales converged, bodies parallel and almost touching, with their flukes beating rapidly and synchronously” (pp 14). Interestingly, sometimes the entire group orientated their bodies in the same direction, usually leaning their left sides toward the surface (Visser et al., 2008), or at other times dividing downwards the middle with the individuals on the right side leaning to the right, and those on the left leaning left (Pitman and Durban, 2012). Sometimes they also produced a coordinated trail of bubbles with their blowholes as they accelerated and passed directly under the ice floe (Visser et al., 2008), in an apparent attempt to mistake or scare the seal (Pitman and Durban, 2012).

Recent controlled assessments of the development of synchrony in new born calves vis-à-vis their mothers highlight the strong predisposition of mother-calf pairs to spend most of their time behaving synchronously (Fellner et al., 2006). Because dolphin calves apparently move continuously for the first month of their lives and stop comparatively infrequently for the first 3 months, the substantial energetic benefit they gain through slipstreaming may provide a mandate for mother-calf synchrony in terms of calf survival. This constant intimate contact may lead to a succession of developmental stages in the calf that proceed from passive to active maintenance of motor synchrony and ultimately to imitation (Fellner et al., 2006). Importantly, in parallel, the calf is learning the signature whistle and that this learning processes is in part also imitative to the mother’s own whistles (see Janik, 2014).

Finally, Cantor et al. (2023) have recently shown that behavioral synchrony plays a significant role in human-cetacean cooperative interactions. The study found that foraging synchrony was the main factor that led to short-and long-term fishing benefits for both humans and dolphins. This synchrony involved both predators coordinating their actions, with dolphins approaching fishermen close to their net and signaling the right moment to cast their nets with a sudden deep dive. This mutualistic foraging tactic between dolphins and humans is thought to be the result of social learning and cultural transmission within and between both species, as suggested by Daura-Jorge et al. (2012).

#### 4.2.2. Vocal synchrony

Acoustic synchrony has been described as a rhythmic repetition of signals in conformity with a regular beat or pulse (Greenfield, 1994). Choruses involve more than two individuals producing a chorus during which there may be some temporally distinct vocal units, and some overlapping vocal units (Baker-Médard et al., 2013). Chorusing by groups can be asynchronous (alternating) or synchronous and does not necessarily involve overlapping vocalizations but instead may have a sequential nature with only slight temporal overlaps of sounds. When synchrony (vocal and physical) is involved in simultaneous calling events, synchrony has been thought to have evolutionary advantages for multi-male groups during various behavior activities (Greenfield, 1994). Synchronized vocal behavior also appears to involve vocal learning and convergence for a few social mammal species including primates and cetaceans (Marler and Mundinger, 1971; Nottebohm, 1976; Boesch, 1991; Janik and Slater, 1997, 2000).

Some instances of vocal chorusing have also been reported in cetaceans, all of them in collaborative contexts. These include the chorusing of the same whistle type in bottlenose dolphins in Scotland (Janik et al., 2011), the rhythmic braying during various social behaviors for bottlenose dolphins (dos Santos et al., 1995; Herzing and Johnson, 2015), and the diving of sperm whales (Whitehead, 1996). Synchronous screams and squawks have been correlated with underwater behavior in Atlantic spotted dolphins, and bottlenose dolphins during intra and interspecific aggression (Herzing, 2005; Herzing and Johnson, 2015). Spotted dolphins were observed using two types of synchronized vocalizations including synchronized squawks (burst pulsed vocalizations) and screams (overlapping FM whistles) during intraspecific and interspecific aggression. Bottlenose dolphins used three types of synchronized vocalizations; whistles/buzz bouts, bray/buzz bouts, and buzz bouts during intraspecific aggression. Importantly body postures were synchronous with physical movements and often mirrored the rhythm of the vocalizations (see Herzing, 2015).

### 4.3. Multimodal and synchronous social learning and communication

Altogether, these observations suggest that social learning, particularly imitation, and synchrony are important communication and cooperative coordination signaling mechanisms for many odontocetes. As mentioned by Stephanie King and McGregor (2016), cetacean vocalizations facilitate social bonding and group synchrony, which makes it comparable to early human communication and other forms of primate vocal communication (like marmoset monkeys turn-taking), and similar to birdsong (see Hage et al., 2016; King and McGregor, 2016; Takahashi et al., 2016; Aboitiz, 2017).

A very interesting example of odontocete multimodal and synchronous social communication in orcas in controlled environments is given by Bowles et al. (2016). She and her collages described in two adult female killer whales two types of synchronous multimodal behaviors that consisted in bouts of stereotyped pulsed calls that occurred concurrently with bubble formations and nodding. As synchronous bubbling behaviors were linked with a functional vocalization type in both subjects, the authors suggested that elements of the vocal repertoire are marked for emphasis, for instance, by a bubble stream. Differential use of synchronous behaviors has a function in call learning. Vocalization rates in other odontocetes increase immediately before parturition and in the presence of calves, after parturition (Mann and Smuts, 1998; Mello and Amundin, 2005; Fripp and Tyack, 2008). This has been interpreted as a behavior that facilitates learning. They suggest that vocalizations are important to calf survival, such as the mother’s signature whistle or primary elements of a group dialect, could be emphasized with synchronous behaviors. This is a very interesting hypothesis that deserves to be explored in the future, not only in the linking between bubbling or other synchronous behaviors with functional vocalization types in the orcas but in other cetaceans as well (Bowles et al., 2016). Supporting these observations, recent experimental evidence suggests that during synchronous behaviors, dolphins use acoustic cues, and more particularly click trains, to coordinate their movements; possibly by eavesdropping on the clicks or echoes produced by one individual leading the navigation (Jaakkola et al., 2018; King et al., 2021; Marulanda et al., 2021).

Finally, although behavioral synchrony can be achieved by other social cognitive mechanisms such as response facilitation by priming (Byrne, 2009), cetaceans also display the capacity to reproduce a demonstrator’s multimodal imitation of novel motor actions and sounds. As mentioned earlier, several odontocetes have synchronous motor behaviors while in parallel learn their distinctive individual and/or group identification calls largely by social learning. This produces several motor and vocal idiosyncratic behaviors from early life that are supported by a multimodal social learning capacity in at least these two domains. Moreover, they also show vocal synchrony in some collaborative contexts. Which instances of motor and vocal social learning are done in a synchronic way remains an open question that certainly deserves attention in the near future.

### 4.4. Multimodal imitation in cetaceans as a comparative model for human language and music evolution

Human language and music are multimodal processes involving not only vocalizations but also facial, hand and body gestures (Arbib, 2011; Cross et al., 2013; Aboitiz, 2017). This supports the notion that there was a close gestural-vocal coevolution in the human lineage (Aboitiz, 2017). There is increasing evidence that communication is multimodal in humans, apes and monkeys. It has been documented that apes can use different channels of communication in an opportunistic way (e.g., Leavens et al., 2004), and it may be that the multimodality that characterizes speech (and singing) has its origins in such capacities (Cross et al., 2013; Aboitiz, 2017). In chimpanzees, Taglialatela et al. (2008, 2011) have shown activation of Broca’s area homolog with the production of both manual and vocal communicative signals. Likewise, Willems and Hagoort (2007) have shown a similar situation in humans. Body gesturing not only accompanies speech, singing (and of course instrumental music), it also helps communication in a similar way as prosody supports speech. It has been pointed out that human communication is opportunistic and multimodal, and that our species uses any possible way to convey messages if we cannot speak (Aboitiz, 2013). Furthermore, speech rhythm is perceived multimodally, using both acoustic and visual cues, from observing lip movements (Peña et al., 2016; Michon et al., 2019, 2020). Importantly, the main point in human speech evolution it is not whether pantomime or vocal behavior was first but that it is only in the human lineage where a combination of learned gestures and vocal plasticity has taken place to develop a symbolic system, possibly fuelled by increasing imitative behavior in both systems.

Supporting this perspective, it has been argued that while birdsong focuses on mate attraction and territorial defense, vocal learning in other animals like parrots and specially cetaceans, promotes social bonds and behavioral coordination, a condition that may be closer to developing a primitive referential system (King and McGregor, 2016). Toothed echolocating cetaceans, probably like our ancestors, live in fission-fusion groups that come together and separate during foraging (Chereskin et al., 2022; Hersh et al., 2022). Their tonal sounds play a vital role in social communication and the sociality of these species has impacted the evolution of tonal sound complexity, as there is a correlation between social and whistle complexity (May-Collado et al., 2007). In large groups, where individuals are far from each other, these species engage in reciprocal vocal exchanges of whistles to establish social bonds, as these sounds replace body contact. Studies have shown that there is a significant correlation between increased tonal sound modulation and group size, and that changes in tonal sound complexity are closely tied to the different social branches of cetaceans (May-Collado et al., 2007).

The complexity that these tonal calls can reach is reflected in the idiosyncratic vocalizations from several cetaceans, which could be seen as proto referential signals that serve to identify group membership and even specific individuals (see Janik and Sayigh, 2013 for review). Field studies of orcas have shown that their call structure (usually referred as dialects) reflects relatedness and social affiliation (Deecke et al., 2010) and sperm whales’, codas may cue individual, unit and vocal clan identity (Schulz et al., 2008; Gero et al., 2016; Oliveira et al., 2016). It has recently been proposed that these “identity codas” may even function as “symbolic” markers of cultural identity among Pacific Ocean sperm whale clans, resembling human ethnolinguistic groups (Hersh et al., 2022). A special and, as far as we know, unique case of referential communication in the non-human animal kingdom, are the dolphins “signature whistles,” learned whistle distinctive to each dolphin, which allows them to recognize one another (see Janik and Sayigh, 2013). However, this signature call is not categorically different from other calls shared by all members of the group but is rather a variant of these. Recent research suggests that dolphin mothers start singing to their babies before they are born, apparently teaching them their signature whistle, a process that continues over the first weeks after birth (King et al., 2016). The signature call of dolphins appears to serve to maintain group cohesion, as individuals separated from the group emit their signature whistles while the others respond with their own signatures until they come together again. Moreover, it has been documented that they transmit identity information independent of the caller’s voice location and even after all voice features have been removed from the whistle signal, something only known to be done by humans (Janik et al., 2006). This is supported by the evidence in the laboratory that bottlenose dolphins can be trained to use novel, learned signals to label objects (Richards et al., 1984) and that they can remember these signature whistles for over 20 years (Bruck, 2013).

These facts support the hypothesis that whistles are referential signals for the representation of conspecifics, that is, they are vocal labels that could be used in a similar way as the use of names in humans, either addressing individuals or referring to them. Furthermore, a recent work has just demonstrated cross-modal, individual recognition not only by sound, but also by taste in bottlenose dolphins (Bruck et al., 2022). In this remarkable study the authors showed that dolphins recognize perception of identity of other individuals by gustation alone and then can integrate information from acoustic and taste inputs to identify specific individuals, indicating a modality independent, labeled concept for known conspecifics. These results show that dolphins form persistent modality-independent multimodal representations that have learned labels similar as in human concept formation (Bruck et al., 2022).

So far, the evidence suggests that vocal learning in cetaceans serves multiple purposes. For instance, the main four proposed functions of humpback song are attracting females to individual singers, determining or facilitating male–male interactions, drawing females to a male group within a lekking system (Herman, 2017), and as a long-range sonar (Frazer and Mercado, 2000; Iii, 2018). Toothed cetaceans primarily use learned vocal signals for maintaining social relationships, group cooperation, and coordination. They also use these signals to identify group membership (such as sperm whale codas and orca dialects) and specific individuals (dolphin signature whistles; see Janik, 2014; Whitehead and Rendell, 2014).

In humans, imitation is also likely to play a major role in the cultural inheritance of communicative and ritualistic behaviors that have an important effect on cooperation within groups (Cross et al., 2013; Heyes, 2021). Nonverbal communicative and ritualistic human behaviors may be targets of cumulative cultural evolution as they may promote group cohesion, supporting forms of cooperation such as long-term teaching and division of labor (Bender et al., 2009). More than grasping behavior, vocal behavior is what has made our lineage different from that of other apes, and probably our vocal system underwent rapid evolution in early stages of our lineage to support social cohesion and behavioral coordination like toolmaking or foraging over long distances. Accordingly, it has been suggested that common proximal factors for humans to communicate through language and music are language’s practical value in the organization of collective behavior and music’s significant role in eliciting and managing prosocial attitudes (Cross et al., 2013). The majority of human speech takes place in back-and-forth conversations. Dunbar (1998) suggests that mutual vocal exchanges in nonhuman primates are akin to grooming behavior, as individuals help each other remove parasites, thereby strengthening their bonds. It is believed that music originally served a similar purpose for human social bonding when group sizes became too large for grooming alone. Our sense of musical tonality may have evolved to recognize and process conspecific vocalizations, which are the most important tonal sound signals in the human environment (Purves, 2017). In this way, music served as a bridge to language as human speech became the primary means of group bonding and cohesion (Dunbar, 2012). This musical protolanguage (Darwin, 1871), which surely included melodic and rhythmic signals expressed through music and dance, was used for social cohesion and behavioral coordination. The melodic contours of early human vocalizations likely emerged as a form of prosody, with changes in pitch and intensity used to convey emotional signals, capture the listener’s attention, and enhance group coordination and cooperation (see Aboitiz, 2017).

Certainly, multimodal imitation in cetaceans convergently serve both motivational functions as well. In fact, similar assumptions have been made for cetacean dialects and other forms of idiosyncratic behaviors. Researchers have highlighted the strong tendency to imitate the actions of members of their own group in several cetacean species, which may fuel intergroup differentiation and intra-group identity (see Byrne, 2009; Whitehead and Rendell, 2014). For instance, it is believed that the dietary choices and foraging behaviors of orcas have a significant cultural component and that social learning plays a role in their development. This learning may involve the use of multimodal imitation for differentiating between different groups and establishing a sense of identity within each group (Barrett-Lennard and Heise, 2006). Certainly, orcas are remarkable conformists about the type of food they eat and the conspecifics with which they interact and reproduce (Baird and Whitehead, 2000; Barrett-Lennard and Heise, 2006). Moreover, evidence of teaching in foraging contexts has been reported in orcas and in Atlantic spotted dolphins (Guinet, 1991; Bender et al., 2009).

## 5. Conclusion

A case can be made that delphinids and other odontocetes possess the capacity to imitate conspecifics’ actions and vocalizations. In the case of humans, evidence has shown that rather than being an exclusively innate ability, imitation capacity develops during infancy and childhood, supported by the maturation of sensorimotor brain networks and domain-general associative learning of multimodal information, both fostered by socially rewarding interactions. This network represents a suitable candidate to coordinate the processing of visual information and the execution of the corresponding motor sequence required for the imitation of facial expressions, such as lip or tongue protrusion (see Michon et al., 2022). Accordingly, recent empirical studies and meta-analyses have challenged the widely assumed view that imitation is an innate skill that emerges independent of environmental contingencies, in both human and non-human primate neonates, indicating other forms of reward-based learning as relevant factors in the development of social behavior (see Heyes, 2021). Recently, Michon et al., 2022 reviewed empirical studies offering new insights from their understanding of speech as the product of evolution and development of a rhythmic and multimodal organization of sensorimotor information, supporting volitional motor control of the upper vocal tract and audio-visual voices-faces integration, and proposed that human imitation relies on crossmodal associations of sensorimotor information (e.g., visuomotor associations for facial imitation and audiomotor associations for vocal imitation) that develop along with social interactions and sensorimotor experience during infancy and childhood.

**Figure 1:**
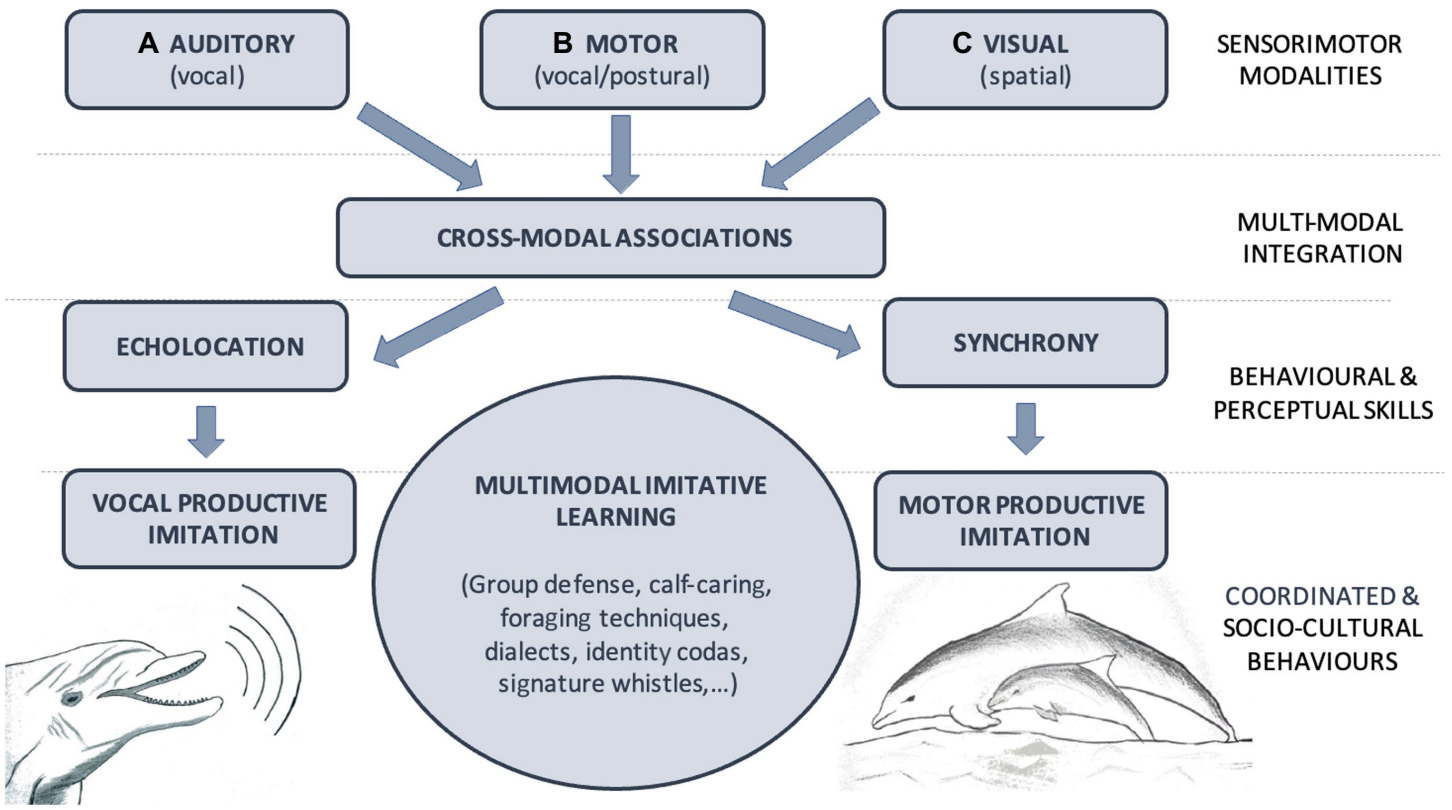
Model for multimodal imitation capacity in cetaceans. Cetaceans uses different *Sensorimotor Modalities* to sense and enact in their environment. **(A)** Auditory system allows the perception of others’ vocalizations and singing (vocal), **(B)** Motor system enable the production of vocalizations, songs and the control of body posture (vocal/postural) while **(C)** Visual system is crucial for the observation of others’ body posture during group behaviors for instance (spatial). Those sensorimotor modalities are integrated by cross-modal associative learning. In individual organism, these multimodal associations underly the consolidation of *Behavioral and Perceptual Skills*, *such as* (a) echolocation and (b) synchrony. Multimodal imitative learning is proposed to be a fundamental mechanism that give rise to complex and idiosyncratic *Coordinated and Socio-Cultural Behaviors* that characterized most cetacean societies.

A similar convergent evolution may have occurred in toothed cetaceans. Their capacity for multimodal imitation is possibly based on cross-modal associations of sensorimotor information (e.g., audiomotor associations for vocal imitation or visuomotor and visuospatial associations for body posture and motor imitation). Two capacities could be responsible for the emergence and/or enhancement of these multimodal associations in cetaceans, both in phylogenetic evolution and ontogenetic development: echolocation and behavioral synchrony. On the one hand, echolocation, being itself a multimodal perceptual system, would facilitate and enhance the active transformation of acoustic information into visuospatial representation. On the other hand, synchronous vocal and sensorimotor experience during the early years of the mother-calf interactions and vocal and motor synchrony in affiliative and cooperative social interactions throughout their lives would facilitate and enhance imitative abilities in both acoustic and kinesthetic domains (see Figure 1). It is an open question whether the same occurs in baleen cetaceans, but this is most likely to be the case, according to the hypothesis that humpback whale songs would also serve the function of a sonar and given that they exhibit motor synchrony in mother-calf coordination during diving and in adult cooperative feeding strategies.

Therefore, cetacean multimodal imitation could had been acquired in parallel with the evolution and development of a synchronic and multimodal organization of sensorimotor information (Michon et al., 2022), supporting volitional motor control of their vocal system and audio-echoic-visual voices and body posture and movement integration. It remains an open question to what degree this multimodal imitative capability depends on simple associative learning mechanisms or a natural predisposition that can be primed and enhanced under certain environmental conditions, for example, when they are involved in naturally occurring social interactions, when they are exposed to ecological demands that require highly synchronous and coordinated group activities such as hunting, or when they participate in training sessions in a controlled setting.

Finally, due cetaceans are one of the few mammals to have shown, so far, the multimodal imitative abilities discussed, they stand out as a model for contributing to the question of one of the major unsolved puzzles of human evolution, that is, how speech and singing evolved and the evolutionary process required for the emergence of human linguistic and musical communication.

## Author contributions

JZ-A took the lead in writing the manuscript. MM, MH-L, and FA provided critical feedback and helped shape the theoretical analysis and the manuscript’s text, figures and tables. All authors contributed to the article and approved the submitted version.

## Funding

This research was supported by grants from ANID Fondecyt Iniciación Folio 11201224 to JZ-A, ANID Fondecyt Postdoctoral Folio 3201057 to MM, and ANID Fondecyt Regular Folio 1210659 to FA.

## Conflict of interest

The authors declare that the research was conducted in the absence of any commercial or financial relationships that could be construed as a potential conflict of interest.

## Publisher’s note

All claims expressed in this article are solely those of the authors and do not necessarily represent those of their affiliated organizations, or those of the publisher, the editors and the reviewers. Any product that may be evaluated in this article, or claim that may be made by its manufacturer, is not guaranteed or endorsed by the publisher.
